# Acupuncture for attention deficit hyperactivity disorder (ADHD): study protocol for a randomised controlled trial

**DOI:** 10.1186/1745-6215-12-173

**Published:** 2011-07-11

**Authors:** Soon-Sang Hong, Seung-Hun Cho

**Affiliations:** 1 Department of Neuropsychiatry, College of Korean medicine, Kyung Hee University, Hoegi-dong Dongdaemun-gu, Seoul, Korea

## Abstract

**Background:**

Attention-deficit/hyperactivity disorder (ADHD) is a common neuro-psychiatric problem, affecting 7-9% of children. Pharmacological interventions are widely used with behavioral treatments in ADHD. Still, the origin of ADHD is unclear, limiting pharmacological effectiveness and making adverse effects common. The use of complementary and alternative medicine (CAM) has increased, especially for developmental and behavioral disorders, such as ADHD. CAM is used by 60-65% of parents of children with ADHD to relieve ADHD-associated symptoms and to avoid the side effects of conventional medication. Acupuncture has been widely used to treat patients with ADHD, but the available evidence of its effectiveness is insufficient. Our aim was to evaluate the effectiveness and safety of acupuncture in patients (both and each treatment naive and conventional therapy children) with ADHD (any subtype) compared to the waitlist control.

**Methods/Design:**

This study is a waitlist controlled open trial. We used a computer generated randomization scheme. This randomised, controlled trial had two parallel arms (acupuncture, and waitlist group). Each arm consisted of 40 participants. The acupuncture group received acupuncture treatment two times per week for a total of 12 sessions over 6 weeks. Post-treatment follow-up was performed 3 weeks later to complement the 12 acupuncture sessions. Participants in the waitlist group did not receive acupuncture treatments during the first six weeks but were only required to be assessed. After 6 weeks, the same treatments given to the acupuncture group were provided to the waitlist group. The primary outcome of this trial included differences in Korean version of ADHD-Rating Scale (K-ADHD-RS) before randomization, 3 weeks and 6 weeks after randomization, and 3 weeks after completing the treatment.

**Discussion:**

Subjective measurements, like K-ADHD-RS, are commonly used in ADHD. Although these measurements have adequate reliability and validity, lack of objective assessment in ADHD may lead to some disputes, like parental placebo effects. More objective measurements, like Computerized Neurocognitive function Test (CNT) in this study, are needed in ADHD trials. Furthermore, this trial will provide evidence for the effectiveness of acupuncture as a treatment for ADHD.

**Trial Registration:**

Clinical Research Information Service (CRiS) KCT0000019

## Background

Attention-deficit/hyperactivity disorder (ADHD) is a common problem, affecting 7-9% of children. The defining features of ADHD are inattention, hyperactivity, and impulsivity [1]. There is an abundance of literature dedicated to research for the treatment of ADHD) [2-6]. Most of these research have focused on pharmacological therapies with less emphasis on psychotherapy and psychosocial interventions [7-9] and even less in the area of complementary and alternative medicine (CAM) [10,11]. However, the origin of ADHD is unclear, which limits pharmacological effectiveness and makes adverse effects common.

The widespread use of stimulant medications for ADHD, such as methylphenidate (MPD) and dex-amphetamine, is thought to enhance short-term behavioral, academic, and social functioning [12]. However, concern about side-effects, such as tics, insomnia, and irritability, as well as questions about the long-term safety of these medicines and personal preference to avoid stimulants, has led many parents to seek alternative treatments for ADHD [13-15].

The use of CAM therapies has increased, especially for developmental and behavioral disorders such as ADHD [10,13,16-18]. Data from family practice physicians and internists show that acupuncture is one the most frequently recommended CAM therapies [19,20]. Approximately 60-65% of parents with children diagnosed with ADHD have used CAM therapies [10,16,17,21,22]. Acupuncture is a relatively simple, inexpensive, and safe treatment compared to other conventional interventions. Acupuncture has been widely used to lessen the severity of the core symptoms in patients with ADHD [23,24]. As a therapeutic intervention, acupuncture is also increasingly practiced in some Western countries [25,26]. However, existing evidence may not be rigorous enough for acupuncture to be recommended for routine use.

Acupuncture can change brain activity [27-35]. A brain hemodynamic study conducted in Japan reported that acupuncture significantly decreased activity in the dorsomedial prefrontal cortex (DMPFC) [29]. Hyperactivity in the DMPFC is associated with various psychiatric diseases with socio-emotional disturbances such as schizophrenia and ADHD. A fluorodeoxyglucose positron emission tomography combined computed tomography (FDG-PET/CT) study in Korea reported changes in brain glucose metabolism in specific brain areas following stimulation by acupuncture [32]. Collectively, these findings suggest that acupuncture might be effective to treat ADHD.

There are a large number of studies of the clinical efficacy of acupuncture in ADHD published globally, especially in China, but not all demonstrate a beneficial effect on ADHD [13,23,24,36].

### Objectives

The present study aimed to evaluate the effectiveness of acupuncture in patients with ADHD (any subtype) compared to waitlist control and to evaluate the safety of acupuncture in patients with ADHD (any subtype).

## Methods/Design

### Design

A 6-week, randomised, waitlist controlled trial of acupuncture for the treatment of ADHD in children/adolescents is being conducted at Kyung-Hee Oriental Medical center, Seoul, Korea. All screening appointments and study visits occur in the neuropsychiatry clinic for outpatients. This study was ethically approved by the Kyung-Hee Korean Medical Center Institutional Review Board, and was registered with the Clinical Research Information Service (CRiS) (KCT0000019) as a waitlist controlled, randomised clinical trial. This study had two parallel arms (acupuncture and waitlist group) with a 1:1 patient allocation ratio (40 participants per arm).

### Care providers

The providers went through 6 years of regular Korean medical college education and each was licensed a Korean Medical Doctor (KMD) degree. All the KMDs have undergone acupuncture safety education. These practitioners were chosen on the basis of their expertise in the field, their certified status and agreement to abide by the study protocols. Every practitioner is an apprentice doctor of "Department of Neuropsychiatry, College of Korean medicine, Kyung-Hee University" during this trial period.

### Participants

Parents were initially screened by phone, and their children were included in the study if the inclusion criteria were met. The inclusion criteria includes being between 7-18 years of age, having a diagnosis of ADHD (any subtype) according to Diagnostic and Statistical Manual of Mental Disorders (DSM-IV) criteria and having an informed verbal and written consent from parents and participants. It did not matter whichever intervention (pharmacological, psychosocial therapy, educational, or occupational therapy, as examples) was used, if there were not any change of ADHD treatments/symptoms for the past 2 weeks. Exclusion criteria are a diagnosis of a developmental disorder, past history of epilepsy, the presence of another neurotic disorder, pregnancy, and any changes in medications during the course of the study. During waitlist period, a CGI-I score ≥6, is a cue for the participant to stop waiting and to drop-out.

### Measures

DSM-IV ADHD diagnostic criteria [1,37] were used by the principal investigator to establish a diagnosis. Once randomised, participants were evaluated by the principal investigator during study visits at baseline and weeks 3 and 6 (or 3, 6, 9, and 12). The reliable and validated Korean version of the ADHD rating scale (K-ADHD-RS) [38] was used pre- and post-intervention as a primary outcome measure.

### Primary outcome measures

The primary outcomes for the study were changes in ADHD symptoms from baseline to week 6 (or from week 6-12) as measured by the K-ADHD-RS [38]. The K-ADHD-RS is an 18-item standardized, valid, reliable instrument for the diagnosis and also a weekly assessment tool for checking the of treatment response among children and adolescents with ADHD [39]. Each item on the instrument describes one of the symptoms of ADHD rated on a 0-3 point Likert scale (0 = never or rarely, 1 = sometimes, 2 = often, and 3 = very often) [39]. The principal investigator administered the K-ADHD-RS to the parent at each study visit. Nationally representative norms are available for the scale and were used to determine eligibility [39].

### Secondary outcomes

The Clinical Global Impression (CGI) rating scale is also a commonly used measure of symptom severity, treatment response, and the efficacy of treatments in treatment studies of patients with mental disorders [40]. The Clinical Global Impression severity scale (CGI-S) was used at week 3 and 6 (or weeks 3, 6, 9, and 12). CGI-S requires the clinician to rate the severity of the patient's illness at the time of assessment, relative to the clinician's past experience with patients who have the same diagnosis. Considering total clinical experience, a patient is assessed on the severity of mental illness at the time of rating on a 1-7 scale (1 = normal or not at all ill, 2 = borderline mentally ill, 3 = mildly ill, 4 = moderately ill, 5 = markedly ill, 6 = severely ill, and 7 = extremely ill) [41]. The Clinical Global Impression improvement scale (CGI-I) is used at week 3 and 6 (or weeks 3, 6, 9, and 12) to rate the worsening, maintenance, or improvement in global impairment of participants enrolled in the study compared with baseline [41]. The CGI improvement scale includes an eight-point scale for scoring (0 = not assessed, 1 = very much improved, 2 = much improved, 3 = minimally improved, 4 = no change, 5 = minimally worse, 6 = much worse, and 7 = very much worse) [41].

The Korean version of Child Behavior Checklist (K-CBCL) was used at week 3 and 6 (or weeks 3, 6, 9, and 12). CBCL has been proposed as a non-ideological measure to characterize comorbidity in individuals with ADHD [42,43]. The CBCL is an empirically derived, highly valid and reliable parent checklist of pediatric psychopathology that is composed of several dimensions and scored according to established algorithms [44]. As a parent-completed measure, the CBCL provides a common metric across patients and studies that is free from clinician interpretation [45]. Subsequent reports have proven the utility of this approach [46-50].

The Frankfurt Attention Inventory (FAIR) [51] measuring concentration behavior consists of 640 stimuli with high similarity that have to be discriminated against each other within 6 min. The FAIR test quantifies aspects of concentration behavior displayed in four test scores: marker value (MV) expresses comprehension of test instructions, performance value (PV) informs on the amount of attentively processed test items during a defined test period, quality value (QV) depicts the amount of attentively made decisions respective to the total amount of decisions, and the continuity value (CV) reflects the extent of continuously upheld concentration over the entire test duration. The FAIR task provides good test-retest reliability expressed as a Crohnbachs' alpha between 0.85 and 0.91 [51].

The Computerized Neurocognitive function Test (CNT) is used at week 3 and 6 (or week 3, 6, and 12). CNT is extremely sensitive to virtually all of the clinical conditions associated with cognitive dysfunction. It is capable of calculating reaction times with millisecond accuracy, and can generate massive amounts of precise data. In this study, CNT40^© ^(Maxmedica; http://www.maxmedica.co.kr, Seoul, Korea)[52] is selected to make cognitive evaluations of attention and memory function. CNT40 consists of 18-items: Digit Span Test, Visual Span Test, Auditory CPT, Auditory Controlled CPT, Visual CPT, Visual Controlled CPT, Crossover Test, Modality Shift Test, Word-Color Test, Trial Making Test, Card Sorting Test, Verbal Learning Test, Visual Learning Test, Hypothesis Formation Test, Finger Tapping Test, Raven's CPM, Raven's SPM and Color Trail Making Test. Among these items, the Digit Span Test, Visual Span Test, Auditory CPT, Visual CPT and Verbal Learning Test are used to assess attention and memory dysfunction in ADHD patients.

Korean version of Conners' parent Rating scale: short version (K-CPRS) [53,54] and Korean version of IOWA-Conners Rating scale: short version (K-IOWA) [55], which use observer ratings, are included to help assess ADHD and evaluate problem behavior in children and adolescents.

### Randomization and treatment allocation

This study is designed as a waitlist controlled open trial. Eligible participants were randomly allocated to one of two groups: group A (acupuncture) and group W (waitlist). In each group, participants were stratified according to the pharmacologic intervention at the initiation of the study. A computer generated randomization scheme was used. The principal investigator generated the random allocation sequences without any notice to sub-investigators. Stratified randomization was performed as a separate randomization procedure within each of two subsets of participants (without pharmacologic intervention, 1-60; with pharmacologic intervention, D1-D40. Other treatments were not concerned.) by using block size "4". Each number and its randomised letter (A or W) were placed in an opaque envelope. Each envelope was numbered consecutively from 1-60 and D1-D40. As each parent called to have their child screened, they were allocated the next consecutive number once they were eligible, so that for each eligible participant, no. 1 was allocated to envelope no. 1, etc. The screener was unaware of the sequence of group allocation at the time of screening phase. Hence, allocation was concealed at the time of recruiting.

### Sample size justification

Sample size calculations are performed to determine the number of participants needed to detect effect sizes similar to those that have been reported in recent ADHD medication trials [56]. This study is powered to detect a 7.04 difference between the groups. A sample size of 32 per group is required to achieve 80% power with a 2-tailed significance level of .05, assuming an equivalent standard deviation of 9.9 in both groups. Estimating a 20% dropout during the study, a minimum of 80 total participants are needed to reach the target 32 participants per group [, μ_c _- μ_t_: Mean difference = 7.04, σ^2 ^= Assumed Common Variance of both treatment and control group (Standard Deviation = 9.9), n = 32]

### Recruitment and withdrawals

Recruitment for the study commenced in July 2010 and the first participant was enrolled in July 2010. Participant recruitment is ongoing. Each participant is qualified and enrolled into the study separately, and has their own start date. The study end-point goal is December 2011. Paid advertisements were placed in free-newspapers with metropolitan-wide circulations. Paid advertising bills were also inserted between the pages of the morning newspaper. Websites advertisements were placed on several blogs for free, including http://www.ebombit.com and happiend.tistory.com. Short, text-only advertisements were also placed on several ADHD parents' internet community sites for free. As part of the informed consent forms, the parents of the participants were advised that they were free to withdraw their child/children from the study at any stage without any consequences.

### Protocol

Group A (acupuncture group) continued with their existing program and the acupuncture protocol was added to their regimen. Group W (waitlist group) continued with their existing program and the waitlist protocol was added to their regimen. The participants in group A attended a clinical facility for the first 6 weeks and received 12 treatments (two times per week) for approximately 20 minutes. During this first 6-week period, group W consisted of children who continued with their existing ADHD treatment regimen as prescribed by their pediatrician, clinical psychologist and/or medical doctor. Then, the participants in group W were scheduled for another 12 treatments over the next 6 weeks (same as group A, two times per week).

### Acupuncture protocol

#### Definition of acupuncture

Acupuncture originated in ancient East Asia approximately 2,500 years ago and is a key component of traditional Korean medicine or traditional Chinese medicine. Acupuncture techniques involve insertion of needles and the stimulation of the needles may involve manual, electrical, heat, laser or other forms of stimulation [57].

#### Acupuncture protocol

For every treatment visit, participants are treated with 13 acupuncture points [Baihui (GV20)*1, Sishencong (EX-HN1)*4, Hegu (LI4)*2, Quchi (LI11)*2, Sanyinjiao (SP6)*2, Taichong (LR3)*2] (Table 1) [58]. Points are first sterilized with alcohol, and then thin, disposable acupunctures are inserted to a depth of approximately 0.3-0.5 B-cun (head: 0.3 B-cun; arms and legs: 0.5 B-cun) until a characteristic de qi is felt by the patient without manual stimulation. Acupunctures are maintained for 20 minutes in a supine position. Stainless steel acupunctures (0.20 mm in diameter and 30 mm long; DONGBANG Acupuncture; http://www.dbneedle.com; Seoul, Korea) are used. This treatment is conducted two times per week for 6 weeks. There are no co-interventions without a life-style education for their parents. An education for parents is conducted during visits in weeks 3 and 6.

**Table 1:** Acupuncture points selected in this protocol

Acupuncture point	location
Baihui (GV20)	On the head, 5 B-cun superior to the anterior hairline, on the anterior median line. Note 1: GV20 is located in the depression 1 B-cun anterior to the midpoint of the line from the anterior hairline to the posterior hairline. Note 2: When the ears are folded, GV20 is located at the midpoint of the connecting line between the auricular apices.
Sishencong (EX-HN1)	EX-HN1 is located four points on the vertex of the head, 1 B-cun anterior, posterior and lateral to GV20.
Hegu (LI4)	On the dorsum of the hand, radial to the midpoint of the second metacarpal bone.
Quchi (LI11)	On the lateral aspect of the elbow, at the midpoint of the line connecting LU5 with the lateral epicondyle of the humerus. Note: When the elbow is fully flexed, LI11 is located in the depression on the lateral end of the cubital crease.
Sanyinjiao (SP6)	On the tibial aspect of the leg, posterior to the medial border of the tivia, 3 B-cun superior to the prominence of the medial malleolus. Note: 1 B-cun superior to KI8.
Taichong (LR3)	On the dorsum of the foot, between the first and second metatarsal bones, in the depression distal to the junction of the bases of the two bones, over the dorsalis pedis artery.

* B-cun: Proportional bone cun. This method divides the height of the human body into 75 equal units. Using joints on the surface of the body as the primary landmarks, the length and width of every body part is measured by such proportions.

#### Waiting list protocol

The control group will be in the 6 weeks waiting list without getting additional ADHD treatment. In the first visit at week 3 and at week 6, a life-style education for parents will be conducted with an ADHD symptom improvement check. A CGI-I score ≥6, is a cue for the participant to stop waiting and drop-out (then, the same as in the acupuncture treatment group for study ethical issues). After the waiting endpoint, participants will similarly be given acupuncture treatment for 6 weeks without an additional life-style education for parents.

### Risks

One of the advantages of acupuncture is that the incidence of adverse effects is substantially lower than that of many drugs used for the same conditions [59]. In a review by Ernst [60], the most common reported adverse effects were pain (1-45%), fatigue (2-41%), and bleeding (0.03-38%). Although these are rare [61,62], acupuncture is associated with life-threatening complications that include pneumothorax, angina pectoris, septic sacroilitis, epidural abscess, and temporomandibular abscess [63]. For an accurate observation of acupuncture adverse effects, we made an "Adverse Events Report" at every visit.

### Ethics review and informed consent

The trial protocol was reviewed by the Kyung-Hee Korean Medical Center Institutional Review Board. We prepared two types of consent forms, one for participants who are 7-13-years-of-age (explained in easier words) and another for those who are 14-18-years-of-age. For 7-13-year-old participants, a "comprehension check form" is used to confirm their full understanding. All children participants make their own decisions about participation in a clinical trial. All parents of the participants are issued with duplicate copies of the informed consent forms, with the original copies kept by the hospital. The informed consent form conveys the following information: names of the chief investigator and supervisors; the name of the hospital and the department conducting the research, a brief description of the control and treatment groups, the duration of the study, the questionnaires used, the Ethics approval, the right to withdraw their child at any time, and the fact that the results of the study will be disseminated via conferences and publications while maintaining the child's privacy.

### Analysis

Tests of potential differences across study groups on demographic and clinical history variables were performed by means of analysis of covariance (ANCOVA) for continuous measures and Pearson χ^2 ^for categorical variables. Every analysis conducted using the SPSS software (SPSS 12.0KO for Windows^©^), and performed among two randomised (acupuncture, and waitlist) groups and their stratified (treatment naive, and conventional therapy) sub-groups. The primary analysis will compare outcomes for each primary and secondary outcome measure among the groups to estimate treatment effect with 95% confidence interval (CI). An intention-to-treat (ITT) analysis will be undertaken of the 3-week and 6-week (respectively 9-week and 12-week) data from the K-ADHD-RS, due to some participants who commence the study but do not complete.

## Discussion

We have a presented the design and the protocol for the randomised controlled trial of acupuncture for childhood ADHD. The participants of this study will have acupuncture added to their existing medical regimen (pharmacologic and/or psychosocial) or no previous therapy at all, and will be monitored for changes in their outcomes (i.e. inattention, hyperactivity and impulsivity) in the short-term (3 and 6 weeks) and during a 9 week follow-up. Completion of this trial will help provide the answer to the question "Does acupuncture have a role to play in the management of children with ADHD?" Results of this trial will be disseminated at the completion of the study.

This is the first acupuncture trial protocol study in ADHD. Case studies of acupuncture effects for ADHD already exist, especially in China, but trial protocols are not precisely described. Our protocol will help to make better quality acupuncture studies for ADHD. Furthermore, we tried to gather objective measurements using CNT40. Subjective measurements, like K-ADHD-RS in this study, are commonly used in ADHD and have adequate reliability and validity. But lack of objective measurements in ADHD may lead to some disputes like parental placebo effects.

In traditional Korean medical theories, the insufficiency of yin, results in an excess of yang. The imbalanced condition of the whole body leads to impaired mental symptoms like attention deficit, hyperactivity and impulsivity. Thirteen acupuncture points have been chosen in this context. The acupuncture points located on the head region takes a main role for controlling the spirit. Qi of five viscera and six bowels belong to the head region. For balance of the whole mind and body, acupuncture points in the head region are essential. Baihui (GV20), Sishencong (EX-HN1) are representative points for the head region. Quchi (LI11) takes a role in the elimination of excessive yang and Sanyinjiao (SP6) supplements insufficient yin. It yields a better mental balance. Hegu (LI4), Taichong (LR3) assists in harmonious circulating qi and blood.

One limitation of this study involves the waitlist for the controlled group. Many researchers commonly prefer placebo controlled designs to the waitlist ones in randomised control trials. Placebo effects of acupuncture were actively studied in many articles and sham acupuncture is a representative method for an acupuncture placebo tool. But there is a lack of consensus on the most valid approach to establishing an acupuncture placebo. So, important aspects of the placebo acupuncture control need to be considered. "Inert" is the main topic in placebo acupuncture control. Placebo acupuncture strategies mainly consist of superficial (or minimal) insertion, non-acupuncture insertion points insertion, and sham acupuncture. But each approach has some defects. First, a superficial (or minimal) insertion is not a real placebo method because it induces peripheral sensory stimulation. It does not seem possible to insert acupuncture needles without any sensory stimulation [64,65]. In fact, even a very mild form of placebo acupuncture seems to exert an effect [66]. Second, non-acupuncture insertion points may not be inert. Needling in the areas around the traditional acupuncture points may be equally efficient [66]. Acupuncture areas or zones are understood to be like acupuncture points in the current ongoing discussion. Third, some articles have suggested that sham acupuncture may not be inert [67,68]. Re-examination of acupuncture trials is needed, to ascertain whether a sham that was assumed to be inert was in fact not inert [67]. In this context, a placebo acupuncture, as a control intervention, is not adequate enough to observe acupuncture effects. Although waitlist is an imperfect control method as well, we used it to observe definite acupuncture effects in ADHD.

Not separating assessor from randomization is an additional limitation. Every measurement was assessed by the person who conducted the screening phase, so as not to be blinded from randomization. Although we tried to maintain an objective standpoint, we could not exclude observer bias, which all researchers brought to their work and which helped to determine their method of research and their observations. To minimize this bias, we involved objective ADHD measurement tools, like CNT40 and FAIR.

Finally, a paper case record form (CRF) was used in this study. These days, electronic case record form (e-CRF) system, like the Clinical Data Interchange Standards Consortium (CDISC) e-CRF, is become preferred because of its improved quality and efficiency in trial study. In July 2010, the Korea CDISC Coordination Committee (K3C) started making standard e-CRF forms, but we used paper CRF instead, due to insufficient e-CRF infrastructure. Paper CRF may lead to data loss and input errors. The Kyung-Hee Korean Medical Center Institutional Review Board strictly supervised data storage and management. We also reexamined the inputted data.

Subjective measurements, like K-ADHD-RS, are commonly used in ADHD, and have the best evidence. Although these measurements have adequate reliability and validity, lack of objective assessment in ADHD may lead to some disputes, like parental placebo effects. More objective measurements, like the presently used CNT40, have to be validated in ADHD trials. Furthermore, as an evidence-based treatment in ADHD, acupuncture has little evidences compared with conventional pharmacological or psychosocial treatment. This study will give some evidence for the effectiveness of acupuncture as an add-on treatment in treating children with ADHD.

## List of abbreviations used

ADHD: Attention Deficit Hyperactivity Disorder; CAM: Complementary and Alternative Medicine; DMPFC: Dorsomedial Prefrontal Cortex; FDG-PET/CT: Fluorodeoxyglucose Positron Emission Tomography combined Computed Tomography; K-ADHD-RS: Korean version of ADHD-Rating Scale; CRiS: Clinical Research Information Service; MPD: methylphenidate; KMD: Korean Medical Doctor; CGI: Clinical Global Impression rating scales; CGI-S: Clinical Global Impression severity scale; CGI-I: Clinical Global Impression improvement scale; K-CBCL: Korean version of Child Behavior Checklist; FAIR: The Frankfurt Attention Inventory; MV: marker value; PV: performance value; QV: quality value; CV: continuity value; CNT: Computerized Neurocognitive function Test; CPT: Continuous Performance Test; CPM: Colored Progressive Matrices; SPM: Standard Progressive Matrices; K-CPRS: Korean version of Conners' parent Rating scale; K-IOWA: Korean version of IOWA-Conners Rating scale; group A: acupuncture group; group W: waitlist group; SD: Standard Deviation; GV: Governor Vessel; EX-HN: Extra Points of Head and Neck; LI: Large Intestine Meridian; SP: Spleen Meridian; LR: Liver Meridian; B-cun: proportional bone cun; WHO: World Health Organization; CI: confidence interval; ITT: Intention-To-Treat; CRF: Case Record Form; e-CRF: Electronic Case Record Form; CDISC: Clinical Data Interchange Standards Consortium; K3C: Korea CDISC Coordination.

## Competing interests

The authors declare that they have no competing interests.

## Authors' contributions

SHC conceived the research project. SHC, SSH designed the study. SSH designed and tailored the protocol for the ADHD cohort. All authors contributed to the writing of the manuscript. All authors read and approved the final manuscript.

## References

[B1] American Psychiatric Association Diagnostic and Statistical Manual of Mental Disorders, Fourth Edition, Text Revision (DSM-IV-TR) 2000 Washington DC: American Psychiatric Association

[B2] B W L G J W Attention-Deficit/Hyperactivity Disorder Textbook of child and adolescent psychiatry 2004 3 M D: American Psychiatric Publishing 485 508

[B3] Dulcan M Practice parameters for the assessment and treatment of children, adolescents, and adults with attention-deficit/hyperactivity disorder. American Academy of Child and Adolescent Psychiatry J Am Acad Child Adolesc Psychiatry 1997 36 85S 121S 9334567 10.1097/00004583-199710001-00007

[B4] DuPaul GJ Assessment of ADHD symptoms: comment on Gomez et al. (2003) Psychol Assess 2003 15 115 117 12674731 10.1037/1040-3590.15.1.115

[B5] Johnston C Leung DW Effects of medication, behavioral, and combined treatments on parents' and children's attributions for the behavior of children with attention-deficit hyperactivity disorder J Consult Clin Psychol 2001 69 67 76 11302279 10.1037//0022-006x.69.1.67

[B6] Naglieri JA Goldstein S Delauder BY Schwebach A Relationships between the WISC-III and the Cognitive Assessment System with Conners' rating scales and continuous performance tests Arch Clin Neuropsychol 2005 20 385 401 10.1016/j.acn.2004.09.008 15797174

[B7] P S Trends in the prescribing of stimulant medication for the treatment of Attention Deficit Hyperactivity Disorder in children and adolescents in NSW 2002 Sydney: NSW Department of Health 10.1071/nb02s01 12189396

[B8] P S Trends in the prescribing of stimulant medication for the treatment of Attention Deficit Hyperactivity Disorder in children and adolescents in NSW 2004 Sydney: NSW Department of Health

[B9] Schachter HM Pham B King J Langford S Moher D How efficacious and safe is short-acting methylphenidate for the treatment of attention-deficit disorder in children and adolescents? A meta-analysis CMAJ 2001 165 1475 1488 11762571 PMC81663

[B10] Chan E The role of complementary and alternative medicine in attention-deficit hyperactivity disorder J Dev Behav Pediatr 2002 23 S37 45 10.1097/00004703-200202001-00007 11875289

[B11] L A Treatment alternatives for attention-deficit/hyperactivity disorder (ADHD) J Atten Disord 1999 3 30 48 10.1177/108705479900300103

[B12] Efron D Jarman F Barker M Methylphenidate versus dexamphetamine in children with attention deficit hyperactivity disorder: A double-blind, crossover trial Pediatrics 1997 100 E6 9382907 10.1542/peds.100.6.e6

[B13] Bussing R Zima BT Gary FA Garvan CW Use of complementary and alternative medicine for symptoms of attention-deficit hyperactivity disorder Psychiatr Serv 2002 53 1096 1102 10.1176/appi.ps.53.9.1096 12221307

[B14] Cala S Crismon ML Baumgartner J A survey of herbal use in children with attention-deficit-hyperactivity disorder or depression Pharmacotherapy 2003 23 222 230 10.1592/phco.23.2.222.32092 12587812

[B15] Doggett AM ADHD and drug therapy: is it still a valid treatment? J Child Health Care 2004 8 69 81 10.1177/1367493504041856 15090116

[B16] Bush G Valera EM Seidman LJ Functional neuroimaging of attention-deficit/hyperactivity disorder: a review and suggested future directions Biol Psychiatry 2005 57 1273 1284 10.1016/j.biopsych.2005.01.034 15949999

[B17] Gross-Tsur V Lahad A Shalev RS Use of complementary medicine in children with attention deficit hyperactivity disorder and epilepsy Pediatr Neurol 2003 29 53 55 10.1016/S0887-8994(03)00027-4 13679122

[B18] Rojas NL Chan E Old and new controversies in the alternative treatment of attention-deficit hyperactivity disorder Ment Retard Dev Disabil Res Rev 2005 11 116 130 10.1002/mrdd.20064 15977318

[B19] Diehl DL Kaplan G Coulter I Glik D Hurwitz EL Use of acupuncture by American physicians J Altern Complement Med 1997 3 119 126 10.1089/acm.1997.3.119 9395701

[B20] Gordon NP Sobel DS Tarazona EZ Use of and interest in alternative therapies among adult primary care clinicians and adult members in a large health maintenance organization West J Med 1998 169 153 161 9771154 PMC1305198

[B21] Sinha D Efron D Complementary and alternative medicine use in children with attention deficit hyperactivity disorder J Paediatr Child Health 2005 41 23 26 10.1111/j.1440-1754.2005.00530.x 15670219

[B22] Stubberfield T Parry T Utilization of alternative therapies in attention-deficit hyperactivity disorder J Paediatr Child Health 1999 35 450 453 10.1046/j.1440-1754.1999.355401.x 10571757

[B23] H L Prof. ZHANG Jia-wei Clinical Study on Acupuncture Treatment of 380 Cases of Infantile Attention-deficit Hyperactivity Shanghai Journal of Acu-Mox 2004 23 23 25

[B24] ZY W M L JL Y Clinical observation of ADHD in children with acupuncture and medicine Chinese Journal of Information on TCM 2006 13 78 79

[B25] NIH Consensus Conference. Acupuncture JAMA 1998 280 1518 1524 10.1001/jama.280.17.1518 9809733

[B26] Kemper KJ Complementary and alternative medicine for children: does it work? Arch Dis Child 2001 84 6 9 10.1136/adc.84.1.6 11124773 PMC1718621

[B27] Bai L Tian J Zhong C Xue T You Y Liu Z Chen P Gong Q Ai L Qin W Dai J Liu Y Acupuncture modulates temporal neural responses in wide brain networks: evidence from fMRI study Mol Pain 2010 6 73 10.1186/1744-8069-6-73 21044291 PMC2989943

[B28] Cho SY Jahng GH Park SU Jung WS Moon SK Park JM fMRI study of effect on brain activity according to stimulation method at LI11, ST36: painful pressure and acupuncture stimulation of same acupoints J Altern Complement Med 2010 16 489 495 10.1089/acm.2009.0395 20423217

[B29] Hori E Takamoto K Urakawa S Ono T Nishijo H Effects of acupuncture on the brain hemodynamics Auton Neurosci 2010 157 74 80 10.1016/j.autneu.2010.06.007 20605114

[B30] Hui KK Marina O Claunch JD Nixon EE Fang J Liu J Li M Napadow V Vangel M Makris N Chan ST Kwong KK Rosen BR Acupuncture mobilizes the brain's default mode and its anti-correlated network in healthy subjects Brain Res 2009 1287 84 103 19559684 10.1016/j.brainres.2009.06.061 PMC3742122

[B31] Liu P Zhang Y Zhou G Yuan K Qin W Zhuo L Liang J Chen P Dai J Liu Y Tian J Partial correlation investigation on the default mode network involved in acupuncture: an fMRI study Neurosci Lett 2009 462 183 187 10.1016/j.neulet.2009.07.015 19595739

[B32] Park MS Sunwoo YY Jang KS Han YM Kim MW Maeng LS Hong YP O JH Chung YA Changes in brain FDG metabolism induced by acupuncture in healthy volunteers Acta Radiol 2010 51 947 952 10.3109/02841851.2010.502541 20735279

[B33] Sakatani K Kitagawa T Aoyama N Sasaki M Effects of acupuncture on autonomic nervous function and prefrontal cortex activity Adv Exp Med Biol 2010 662 455 460 10.1007/978-1-4419-1241-1_65 20204829

[B34] Takamoto K Hori E Urakawa S Sakai S Ishikawa A Kohno S Ono T Nishijo H Cerebral hemodynamic responses induced by specific acupuncture sensations during needling at trigger points: a near-infrared spectroscopic study Brain Topogr 2010 23 279 291 10.1007/s10548-010-0148-8 20502956

[B35] Zhang RJ Song XG Cai XH [Influence of acupuncture and moxibustion on conditional position preference and prefrontal cortical ultrastructure in heroin re-addicted rats] Zhen Ci Yan Jiu 2009 34 97 100 19685722

[B36] TQ C XS L Observation on Therapeutic Effects of 155 Cases of Child Attentional Deficit Hyperactivity Disorder Treated with Acupuncture and Moxibustion Chinese Acupuncture and Moxibustion 1999 1 5 6

[B37] American Psychiatric Association Diagnostic and Statistical Manual of Mental Disorders, Fourth Edition(DSM-IV) 1994 Washington DC: American Psychiatric Association

[B38] So YK Noh JS Kim YS Ko SG Koh Y-J The Reliability and Validity of Korean Parent and Teacher ADHD Rating Scale J Korean Neuropsychiatr Assoc 2002 41 283 289

[B39] G D T P A A R R ADHD Rating Scale-IV: Checklists, Norms, and Clinical Interpretation 1998 New York: Guilford Press

[B40] Guy W ECDEU Assessment Manual for Psychopharmacology Rockville, Maryland US Department of Health, Education, and Welfare 1976 217 222

[B41] National Institute of Mental Health Clinical global impressions Psychopharmacol Bull 1985 21 839 843

[B42] Biederman J Monuteaux MC Kendrick E Klein KL Faraone SV The CBCL as a screen for psychiatric comorbidity in paediatric patients with ADHD Arch Dis Child 2005 90 1010 1015 10.1136/adc.2004.056937 16177156 PMC1720123

[B43] Biederman J Wozniak J Kiely K Ablon S Faraone S Mick E Mundy E Kraus I CBCL clinical scales discriminate prepubertal children with structured interview-derived diagnosis of mania from those with ADHD J Am Acad Child Adolesc Psychiatry 1995 34 464 471 10.1097/00004583-199504000-00013 7751260

[B44] TM A Manual for the Child Behavior Checklist/4-18 and 1991 profile 1991 Burlington University of Vermont, Department of Psychiatry

[B45] Mick E Biederman J Pandina G Faraone SV A preliminary meta-analysis of the child behavior checklist in pediatric bipolar disorder Biol Psychiatry 2003 53 1021 1027 10.1016/S0006-3223(03)00234-8 12788247

[B46] Althoff RR Rettew DC Faraone SV Boomsma DI Hudziak JJ Latent class analysis shows strong heritability of the child behavior checklist-juvenile bipolar phenotype Biol Psychiatry 2006 60 903 911 10.1016/j.biopsych.2006.02.025 16650832

[B47] Dienes KA Chang KD Blasey CM Adleman NE Steiner H Characterization of children of bipolar parents by parent report CBCL J Psychiatr Res 2002 36 337 345 10.1016/S0022-3956(02)00019-5 12127602

[B48] Faraone SV Althoff RR Hudziak JJ Monuteaux M Biederman J The CBCL predicts DSM bipolar disorder in children: a receiver operating characteristic curve analysis Bipolar Disord 2005 7 518 524 10.1111/j.1399-5618.2005.00271.x 16403177

[B49] Geller B Warner K Williams M Zimerman B Prepubertal and young adolescent bipolarity versus ADHD: assessment and validity using the WASH-U-KSADS, CBCL and TRF J Affect Disord 1998 51 93 100 10.1016/S0165-0327(98)00176-1 10743842

[B50] Hazell PL Lewin TJ Carr VJ Confirmation that Child Behavior Checklist clinical scales discriminate juvenile mania from attention deficit hyperactivity disorder J Paediatr Child Health 1999 35 199 203 10.1046/j.1440-1754.1999.t01-1-00347.x 10365361

[B51] H M J O FAIR Frankfurter Aufmerksamkeitsinventar 1996 Göttingen: Testmanual

[B52] Lee JB Kim JS Suh WS Sin HJ Bae DS Lee HR The Validity and Reliability of 'Computerized Neurocognitive Function Test' in the Elementary School Child Korean journal of psychosomatic medicine 2003 11 97 117

[B53] Chang MS The Validation of Conners' Adult ADHD Scale-Korean (Short Version) The Korean Journal of Clinical Psychology 2008 27 499 513

[B54] Oh KJ Lee HR Assessment of ADHD with Abbreviated Conners Rating Scale The Korean Journal of Clinical Psychology 1989 8

[B55] Shin MS Ryu ME Kim BN Hwang JW Cho SC Development of the Korean Version of the IOWA Conners Rating Scale Journal of the Korean Neuropsychiatric Association 2005 44 82 88

[B56] Wilens TE Hammerness P Utzinger L Schillinger M Georgiopoulous A Doyle RL Martelon M Brodziak K An open study of adjunct OROS-methylphenidate in children and adolescents who are atomoxetine partial responders: I. Effectiveness J Child Adolesc Psychopharmacol 2009 19 485 492 10.1089/cap.2008.0125 19877972 PMC2861954

[B57] Li S Yu B Zhou D He C Kang L Wang X Jiang S Chen X Acupuncture for attention-deficit hyperactivity disorder (ADHD) in children and adolescents The Cochrane Library 2009 10.1002/14651858.CD007839.pub2 PMC12147681 21491402

[B58] WHO Standard Acupuncture Point Locations in the Western Pacific Region 2008 WHO Regional Office for the Western Pacific

[B59] Breuner CC Complementary medicine in pediatrics: a review of acupuncture, homeopathy, massage, and chiropractic therapies Curr Probl Pediatr Adolesc Health Care 2002 32 353 384 10.1016/S1538-5442(02)90010-6 12486401

[B60] Ernst E White AR Prospective studies of the safety of acupuncture: a systematic review Am J Med 2001 110 481 485 10.1016/S0002-9343(01)00651-9 11331060

[B61] Ernst E White A Acupuncture: safety first BMJ 1997 314 1362 9161302 10.1136/bmj.314.7091.1362 PMC2126657

[B62] Ernst E White AR Indwelling needles carry greater risks than acupuncture techniques BMJ 1999 318 536 10024275 10.1136/bmj.318.7182.536 PMC1114980

[B63] White PF Are nonpharmacologic techniques useful alternatives to antiemetic drugs for the prevention of nausea and vomiting? Anesth Analg 1997 84 712 714 9085944 10.1097/00000539-199704000-00002

[B64] Lewith GT Vincent C On the evaluation of the clinical effects of acupuncture: a problem reassessed and a framework for future research J Altern Complement Med 1996 2 79 90 discussion 91-100 10.1089/acm.1996.2.79 9395647

[B65] Vincent CA Richardson PH The evaluation of therapeutic acupuncture: concepts and methods Pain 1986 24 1 13 3513094 10.1016/0304-3959(86)90022-9

[B66] Mann F Filshie J, White A A new system of acupuncture Medical Acupuncture: A Western Scientific Approach 1998 London: Churchill Livingstone

[B67] Birch S Hesselink JK Jonkman FA Hekker TA Bos A Clinical research on acupuncture. Part 1. What have reviews of the efficacy and safety of acupuncture told us so far? J Altern Complement Med 2004 10 468 480 10.1089/1075553041323894 15253851

[B68] Lund I Lundeberg T Are minimal, superficial or sham acupuncture procedures acceptable as inert placebo controls? Acupunct Med 2006 24 13 15 10.1136/aim.24.1.13 16618044

